# Supplementary material for: Health Status of Mytilus chilensis from Intensive Culture Areas in Chile Assessed by Molecular, Microbiological, and Histological Analyses
Source: Pathogens. 2022 Apr 21;11(5):494. doi: 10.3390/pathogens11050494 (PMC9145640; doi:10.3390/pathogens11050494)
Supplement: Supplementary file 1 [file pathogens-11-00494-s001.zip › pathogens-1493372-supplementary.pdf]

pathogens

MDPI

Supplementary Material

Table S1. Primers sequences used in this study.

Target	Name	Sequence 5 -> 3	PCR product bp	Ref
Marteilia refringens	Pr4	CCGCACACGTTCTTCACTCC	412	[22]
	Pr5	CTCGCGAGTTTCGACAGACG		
B. exitiosa / B. ostrae	Bo	CATTTAATTGGTCGGGCCGC	300	[58]
	Boas	CTGATCGTCTTCGATCCCCC		
Candidatus Xenohaliotis californiensis	RA 3.6	ACTTGGACTCATTCAAAAGCGGA	160	[67]
	RA 5-1	GTTGAACGTGCCTTCAGTTTAC		
P. marinus/ P. olseni	PerKITS85	CCGCTTTGTTTGGATCCC	703	[68]
	PerKITS750	ACATCAGGCCTTCTAATGATG		
OsHV-1	C5	CCGTGACTTCTATGGGTATGTCAG	765	[56]
	C13	CCTCGAGGTAGCTTTTGTCAAG		
	C2	CTCTTTACCATGAAGATACCCACC	352	
	C4	GCAGTTGTGGTATACTCGAGATTG		
HVAb	ORF77F1	CAACCACTTGTTCGGGTTCT	190	[69]
	ORF77R1	CAGGGTGATTAATGCGGAGT		
	ORF77 Probe	FAM-TCCGTACGCGGGATCTTCGT -TAMRA		
Mytilus chilensis	Me15	CCAGTATACAAACCTGTGAAGA	126	[70]
	Me16	TGTTGTCTTAATAGGTTTGTAAGA		
Eukaryotic V4 region	E27F	TCGTCGGCAGCGTCAGATGTGTATAAGAGA-CAGCYGCGGTAATTCCAGCTC		[71]
	E1009R	GTCTCGTGGGCTCGGAGATGTGTATAAGAGA-CAGAYGGTATCTRATCRTCTTYG		

Table S2. Primers concentration and PCR conditions used in this study.

Primer Fwd/Rv	Primer µM	Probe µM	Annealing Tm °C	Time (Sec)	N° Cycles
Bo / Boas	1.0		55	60	35
Pr4 / Pr5	1.0		55	60	40
ORF77F1 / ORF77R1 / Probe	0.3	0.1	60	30	40
RA 3.6 / RA 5.1	0.3		62	60	30
C2 / C6	0.2		50	60	35
PerKITS86 / PerKITS750	0.1		55	60	40
Me15/Me16	0.1		56	30	30

Table S3. Relative abundance (%) of sequences in gut assigned to different families.

**Taxa**	**WT1**	**WT2**	**MF1**	**MF2**	**MF3**
Pteriomorphia	68.8551154	65.9160075	78.0093741	79.0261738	76.2784822
Not_assigned	20.3056609	20.1256088	20.8194826	20.7619244	22.4752859
Peridiniales	7.61998297	12.218167	0.07290959	0.0360853	0.3309753
Copepoda	0.98744988	0.00402961	0.00970951	0.03910566	0.21807799
Mediophyceae	0.55033979	0.45208408	0.02471511	0.00524588	0.0580423
Ebriacea	0.33119192	0.42713886	0.00123576	0	0.00939413
Coscinodiscophytina	0.25037002	0.16214391	0	0	0
Bacillariophyceae	0.1389188	0.0544957	0.00070615	0	0.00553583
Melosirids	0.13220011	0.08961089	0	0	0
Thoracosphaeraceae	0.12350534	0.07330056	0.0164179	0	0.07649505
Syndiniales	0.12073882	0.04777968	0.06320008	0.01859903	0.17932721
Chlorophyceae	0.08576211	0.04566893	0	0	0
Sphaeropleales	0.07904342	0.07234113	0	0	0
Diatomea	0.06382756	0.04048801	0	0	0
Trebouxiophyceae	0.04920453	0.05257684	0	0	0.00402606
Cryptomycota	0.04367149	0.02264258	0	0	0
Bacillariophytina	0.03576715	0.01765354	0	0	0
Collodaria	0.0324078	0	0	0	0
Thecofilosea	0.0278628	0.0055647	0.0019419	0	0.00218078
Suessiaceae	0.02647954	0	0.00547263	0.00238449	0.04227358
Phragmoplastophyta	0.0162039	0	0	0	0
Prasinophytae	0.01561107	0.01592656	0	0	0
Abeoformidae	0.0114613	0.00940243	0.00441341	0	0.00989739
Fragilariales	0.0114613	0.00307018	0	0	0
Pyramimonadales	0.00968282	0	0	0	0

Monogononta	0.00790434	0.00364584	0.03618999	0	0.02516285
Dipodascaceae	0.00770673	0	0	0	0
Gymnodinium_clade	0.00731152	0.00307018	0	0	0
Emiliania	0.00592826	0.00115132	0	0	0.00570358
Oligohymenophorea	0.00592826	0	0	0	0
Chromulinales	0.004545	0	0	0	0
Olpidiopsis	0.00434739	0	0	0	0
Myrtales	0.00414978	0	0	0	0
Pseudoperkinsidae	0.00414978	0	0	0.00222553	0.00603908
Ceratostomataceae	0.00395217	0	0	0	0
Ophiocordycipitaceae	0.00395217	0	0	0	0
Saccharomycetaceae	0.00355695	0	0	0	0
Colpodellida	0.00335935	0.00671602	0	0	0
Paulinellidae	0.00296413	0.00230264	0	0	0
Thraustochytriaceae	0.00256891	0.00172698	0	0	0.0035228
Neopterygii	0.00197609	0	0	0	0
Prostomatea	0.00177848	0	0	0	0
Hydroidolina	0	0.00076755	0.44875586	0.10014863	0.14409923
Gastropoda	0	0	0.25050534	0	0
Thecostraca	0	0	0.09144592	0	0
Scolecida	0	0.00345395	0.05949281	0	0
Heteroconchia	0	0	0.04219223	0	0.02734363
Araucariales	0	0.02916671	0.01659443	0	0
Spumellaria	0	0	0.00847375	0	0
Chromadorea	0	0.01554279	0.00564917	0	0
Palpata	0	0.00479716	0.00476649	0	0
Tetrapoda	0	0	0.0019419	0	0.00067101
Rhodymeniophycidae	0	0.00345395	0.00176537	0	0
Protaspidae	0	0	0.00141229	0	0.02482734
Echinodermata	0	0	0.00123576	0	0
Apiosporaceae	0	0	0	0	0.0070456
Ascidiacea	0	0.01228072	0	0	0
Basal_Group_T	0	0.00249452	0	0	0
Capsasporidae	0	0	0	0	0.00134202
Chlamydomonadales	0	0.00019189	0	0	0
Chlorodendrales	0	0	0	0	0.03103418
Choreotrichia	0	0.00402961	0	0	0
Chytridiomycetes	0	0.00307018	0	0	0
Clade_L	0	0.00057566	0	0	0
Cyphoderiidae	0	0.00767545	0	0	0

Eugregarinorida	0	0.00211075	0	0	0
eukaryote_marine_clone_ME1-24	0	0	0	0	0.00285179
Eustigmatales	0	0.00076755	0	0	0
Glissomonadida	0	0	0	0.00063586	0
Incertae_Sedis	0	0.0209156	0	0	0
Ochromonadales	0	0.00057566	0	0	0
Peregriniidae	0	0.00076755	0	0	0
Peridiniphycidae	0	0	0	0	0.02902115
Perkinsidae	0	0.00153509	0	0	0
Pezizomycotina	0	0.00882677	0	0	0
Sorodiplophrys	0	0.00095943	0	0	0
Spermatophyta	0	0.00172698	0	0	0
Spirotrichea	0	0	0	0.00747141	0
uncultured_eukaryote	0	0	0	0	0.00134202

Table S4. Relative abundance (%) of sequences in gut assigned to different genera.

**Taxa**	**WT1**	**WT2**	**MF1**	**MF2**	**MF3**
Mytiloida	68.8541274	65.9160075	78.0093741	79.0261738	76.2784822
Not_assigned	20.5198686	20.3244029	20.8553195	20.7619244	22.4967582
Heterocapsa	7.42474573	12.0136163	0	0	0
Calanoida	0.90346625	0.00402961	0.00970951	0.02305008	0.21807799
Ebria	0.33119192	0.42713886	0.00123576	0	0.00939413
Thalassiosira	0.33040148	0.30394787	0	0.00381519	0.03657
Actinocyclus	0.17270987	0.11858572	0	0	0
Amphidiniopsis	0.11955317	0.07464376	0	0	0
Syndiniales_Group_I	0.10611579	0.02532899	0.06320008	0.0178042	0.17932721
Cyclotella	0.09900188	0.09805389	0	0	0
Navicula	0.09603775	0.01093752	0	0	0.00553583
Melosira	0.0855645	0.07713828	0	0	0
Monstrilloida	0.07034864	0	0	0	0
Scrippsiella	0.06817495	0.04662837	0.0164179	0	0.06257161
Coscinodiscophytina	0.06382756	0.04048801	0	0	0
Minutocellus	0.06204908	0.03338821	0	0	0
Coscinodiscus	0.05651604	0.02974237	0	0	0
Aulacoseira	0.04663562	0	0	0	0
LKM11	0.04367149	0.02264258	0	0	0
Bacillariophyceae	0.03576715	0.01765354	0	0	0
Siphonosphaera	0.0324078	0	0	0	0
uncultured_Chlorophyta	0.03023411	0.00441338	0	0	0
Chlamydomonas_sp._NIES-3904	0.0300365	0.03204501	0	0	0

Pfiesteria	0.02904846	0.0097862	0	0	0
Coelastrum	0.02746759	0.02225881	0	0	0
Desmodesmus	0.02608433	0.03108558	0	0	0
Symbiodinium	0.02529389	0	0	0	0
Archaeperidinium	0.02470107	0.0195724	0.07290959	0.0360853	0.26739717
Rhaphoneis	0.02430585	0	0	0	0
Durinskia	0.02193455	0.02974237	0	0	0
Actinoptychus	0.02114411	0.01381581	0	0	0
Zygnematophyceae	0.0162039	0	0	0	0
Peridinium	0.01561107	0.00921054	0	0	0
Pseudo-nitzschia	0.01561107	0.04010423	0	0	0
Cyclopoida	0.01363499	0	0	0	0
Diplopsalis	0.01343738	0.02494522	0	0	0
Chaetoceros	0.01185651	0.00575659	0.00300112	0	0.00872312
Fragilaria	0.0114613	0.00307018	0	0	0
Marine_Ichthyosporeans_1	0.0114613	0.00940243	0	0	0
uncultured_eukaryote	0.01027564	0.0237939	0	0	0.00218078
Pterosperma	0.00968282	0	0	0	0
Syndiniales_Group_II	0.00869478	0.00537282	0	0.00079483	0
Ploimida	0.00790434	0.00364584	0.03618999	0	0.02516285
Saprochaete	0.00770673	0	0	0	0
uncultured	0.00632347	0.01036186	0.01041566	0	0.00218078
Emiliania_huxleyi_CCMP1516	0.00592826	0.00115132	0	0	0.00570358
Scuticociliatia	0.00592826	0	0	0	0
Golenkinia_sp._UTEX_931	0.0047426	0.00249452	0	0	0
Chromulina	0.004545	0	0	0	0
Eucalyptus	0.00414978	0	0	0	0
Pseudoperkinsus	0.00414978	0	0	0.00222553	0.00167752
Ophiocordyceps	0.00395217	0	0	0	0
Sphaerodes	0.00395217	0	0	0	0
Saccharomyces	0.00355695	0	0	0	0
Colpodella	0.00335935	0	0	0	0
Paulinella	0.00296413	0.00230264	0	0	0
Mychonastes	0.0023713	0	0	0	0
uncultured_Trebouxiophyceae	0.00217369	0	0	0	0
Teleostei	0.00197609	0	0	0	0
Cryptocaryon	0.00177848	0	0	0	0
Biecheleria	0.00118565	0	0	0	0
Chytriodinium	0.00098804	0	0	0	0
aca	0	0	0.00441341	0	0.00989739

Amphora	0	0.00287829	0	0	0
Anthoathecata	0	0.00076755	0.02065477	0	0
Aplanochytrium	0	0	0	0	0.0035228
Arthrinium	0	0	0	0	0.0070456
Blastodinium	0	0.00172698	0	0	0
Caenogastropoda	0	0	0.14528956	0	0
Choreotrichia	0	0	0	0.00747141	0
Choricystis	0	0.01918863	0	0	0
Chromadorida	0	0	0.00564917	0	0
Cyphoderia	0	0.00767545	0	0	0
D2P04A09	0	0.00076755	0	0	0
D3P05A02	0	0.00211075	0	0	0
Dinobryon	0	0.00057566	0	0	0
Dothideomycetes	0	0.00882677	0	0	0
Echinoidea	0	0	0.00123576	0	0
eukaryote_marine_clone_ME1-24	0	0	0	0	0.00285179
Eunicida	0	0	0.00476649	0	0
Gracilaria	0	0.00345395	0	0	0
Harpacticoida	0	0	0	0.01605557	0
Heterobranchia	0	0	0.10521577	0	0
Heteromita	0	0	0	0.00063586	0
Hyalodiscus	0	0.00076755	0	0	0
Islandinium	0	0	0	0	0.01576872
Leptothecata	0	0	0.42810108	0.10014863	0.14409923
Mammalia	0	0	0.0019419	0	0.00067101
Marine_Group	0	0	0	0	0.00134202
Microglena	0	0.00019189	0	0	0
Monhysterida	0	0.01055375	0	0	0
Monoraphidium	0	0.00172698	0	0	0
Myoida	0	0	0.02701009	0	0
Nannochloropsis	0	0.00076755	0	0	0
Palpata_Incertae_Sedis	0	0.00479716	0	0	0
Parvilucifera	0	0.00153509	0	0	0
Pelagodinium	0	0	0.00547263	0.00238449	0.04227358
Peridiniales	0	0	0	0	0.02902115
Phycodrys	0	0	0.00176537	0	0
Pinophyta	0	0.00172698	0	0	0
Protaspis	0	0	0.00141229	0	0.02482734
Protoperidinium	0	0	0	0	0.04780941
Sessilia	0	0	0.07290959	0	0

Skeletonema	0	0	0.02171399	0.00143069	0.00570358
Spionida	0	0.00307018	0.05949281	0	0
Stephanopyxis	0	0.01170506	0	0	0
Stolidobranchia	0	0.01228072	0	0	0
Tetraselmis	0	0	0	0	0.03103418
Triceratium	0	0	0	0	0.0070456
Tylenchida	0	0.00498904	0	0	0
Uncultured_Cercozoa	0	0.00249452	0	0	0
Uncultured_Chlorella	0	0.00230264	0	0	0
Uncultured_Eimeriidae	0	0.00057566	0	0	0
Uncultured_labyrinthulid	0	0.00095943	0	0	0
Veneroida	0	0	0.01518214	0	0.02734363
Westella	0	0.00287829	0	0	0
